# What Key Factors Affect Patient Satisfaction on Online Medical Consultation Platforms? A Case Study from China

**DOI:** 10.3390/healthcare13050540

**Published:** 2025-03-03

**Authors:** Feng Yang, Yuexin Cheng, Ruiyang Yao, Xiaoqian Zhang

**Affiliations:** 1 School of Public Administration, Sichuan University, Chengdu 610065, China; yangfeng@scu.edu.cn (F.Y.); yaory0909@163.com (R.Y.); 2 School of Information Studies, McGill University, Montreal, QC H3A 1X1, Canada; xiaoqian.zhang@mail.mcgill.ca

**Keywords:** online medical consultation platform, grounded theory, patient satisfaction, DEMATEL, key factors, health information

## Abstract

**Background/Objectives**: Online medical consultation (OMC) platforms have become an essential tool for facilitating communication between doctors and patients, providing an efficient way for patients to access healthcare services. However, research on the key drivers of patient satisfaction within this context remains limited. This study aims to identify and prioritize the key factors influencing patient satisfaction on OMC platforms, with a focus on the Chinese “Chunyu Doctor” app as a case study. **Methods**: Data from patient comments on the “Chunyu Doctor” app were collected and analyzed using grounded theory to identify the influencing factors of patient satisfaction. The decision-making trial and evaluation laboratory (DEMATEL) method was then applied to assess and prioritize the factors influencing patient satisfaction, identifying the key determinants from a complex set of potential influences. **Results**: The study identified 11 key factors out of 23 that significantly impact patient satisfaction. These factors include doctors provide professional treatment plans, doctors accurately understand patients’ concerns, doctors explain and advise on prescriptions, doctors personally respond, doctors provide comprehensive replies, cost-effectiveness, consultation fees, effectiveness of treatment outcomes, reasonableness of the doctors’ consultation process, avoidance of templated responses by doctors, and alignment of doctors responses with patient expectations. **Conclusions**: This study enriches the understanding of patient satisfaction in the context of online medical consultations. The findings offer theoretical insights for future research and provide practical implications for enhancing the management and development of OMC platforms, improving the quality of healthcare services, and boosting patient satisfaction.

## 1. Introduction

With the aging population and the significant increase in life expectancy [1], the demand for healthcare services has risen substantially, leading to greater societal expectations. In the future, online medical and health services are anticipated to enter a new phase of industry transformation, driven by demand [2,3,4]. Given the increasing healthcare needs, there is an urgent necessity to allocate resources equitably and efficiently. Leveraging the rapid advancements in internet technology, online medical consultation (OMC) platforms have proliferated. According to the Global Market Estimates report, the global market for online healthcare consultations is projected to reach USD 16 billion by 2026 [5]. These platforms have brought about significant changes in the healthcare industry, serving as essential mediums for communication and interaction between doctors and patients. Specifically, OMC platforms provide a professional and reliable channel for patients to access health information conveniently [6], thereby helping reduce the uncertainty associated with decision-making. These platforms have become a crucial avenue for patients to obtain healthcare services. Through OMC platforms, doctors can offer diagnostic and treatment advice remotely via various communication methods, including text, phone, or video consultations. Additionally, these platforms provide doctors with more flexibility in selecting patients, allowing them to consider factors such as the severity of the patient’s condition and their relevant medical specialty [7]. Despite the considerable benefits associated with OMC platforms, several challenges remain, including legal, financial, and ethical concerns, as well as ongoing debates regarding their effectiveness and utility [8,9,10,11]. Consequently, the development of more robust OMC platforms and the enhancement of the quality of online consultation services have become critical areas of research [12,13]. In this context, examining patient satisfaction is vital for improving the quality of healthcare services and ensuring the continued advancement of OMC platforms.

Patient satisfaction has been identified as a key indicator influencing patients’ decisions to seek medical advice, comply with prescribed treatment plans, and evaluate the doctor–patient relationship positively [14]. However, patient satisfaction remains a concept characterized by ambiguity, with no consensus on its precise definition or the factors that influence it [15,16]. Numerous studies have examined various determinants of patient satisfaction, such as physician voice characteristics [17,18], physician–patient communication [19], patients’ psychological expectations [20], service quality and pricing [21], and responsiveness and interaction efficiency [22]. Despite this, most existing research focuses on constructing patient satisfaction indicators through literature reviews, expert interviews, and questionnaire surveys. These approaches, while valuable, fail to fully capture patients’ immediate experiences and perceptions within the context of online medical consultation (OMC) platforms.

Patient-generated online reviews have garnered significant attention in the healthcare literature as a visible reflection of patient satisfaction. Analyzing these reviews can help identify the key concerns of patients and clarify the components that contribute to their overall satisfaction with medical care [23,24,25,26]. For instance, Xu (2023) extracted key themes from patient reviews, such as service attitudes, diagnostic accuracy, waiting time, service duration, the insurance reimbursement process, physician skill level, and the hospital environment [27]. Beyond the content of these reviews, their impact on doctor–patient behavior has also been a subject of investigation. Emmert (2022) explored whether physicians modify their service quality in response to patient feedback, revealing that 54% of doctors engage in self-reflection, with improvements primarily observed in communication skills, appointment procedures, and workflow [28]. Similarly, Liu (2021) found that doctors on online health platforms influence the volume, effort, and emotional tone of patient reviews [29]. Grabner-Kräuter and Waiguny (2020) demonstrated that both the number of online doctor reviews and their style significantly affect patients’ willingness to select a particular doctor [30]. In a related study, Lu and Wu (2022) analyzed the information presented on doctors’ homepages across the Good Doctor and Wei-Medicine OMC platforms. Their findings indicated that patients prioritize the number of reviews over the doctor’s overall rating when making their selection [31].

In conclusion, patient comments provide a direct reflection of patients’ perceptions and experiences during the consultation process, and they have a significant influence on the behavior of both healthcare providers and patients. A systematic analysis of these comments is crucial for identifying the challenges within online medical consultation (OMC) services and for implementing targeted improvements. Despite the growing body of literature on OMC platforms, research into patient satisfaction remains fragmented. Existing studies primarily focus on identifying general factors that influence satisfaction, often overlooking the relative importance of these factors and failing to pinpoint the key determinants of patient satisfaction. Furthermore, while patient comments provide a rich source of data that can offer insights into the real needs of users, there is a lack of systematic and in-depth analysis of these comments in the context of OMC platforms.

This study addresses gaps in the existing literature by systematically identifying and prioritizing the key factors that influence patient satisfaction on OMC platforms. Specifically, the research uses the “Chunyu Doctor” app as a case study, extracting relevant patient comments through grounded-theory methodology. By making full use of patient comment data, the study more effectively extracts valuable insights into patient satisfaction. This approach allows for the construction of a comprehensive framework of influencing factors, which represents a departure from previous research that often presents these factors in a fragmented or oversimplified manner. Subsequently, the decision, experiment, and evaluation (DEMATEL) method is applied to identify the key driving factors among the various determinants. By addressing the gaps in the existing literature, this research not only provides a deeper understanding of the dynamics of patient satisfaction in OMC platforms but also offers practical strategies from three perspectives: doctors, patients, and platforms. These strategies aim to improve platform operations, enhance healthcare quality, and increase patient satisfaction. The findings will serve as a theoretical foundation for future research on patient satisfaction in the OMC context and offer actionable insights for improving service quality in online medical consultations.

## 2. Materials and Methods

### 2.1. Data Source

Data were collected from “Chunyu Doctor” (https://www.chunyuyisheng.com/ (accessed on 15 March 2024)), which was launched in 2011 and pioneered online medical consultation in China. As of December 2024, more than 680,000 practicing physicians from public hospitals have settled on the platform, serving more than 400 million patients. Chunyu Doctor has a strong reputation and influence on China’s OMC platform, and it is currently one of the most popular and representative online medical communities. “The Chunyu Doctor” app provides 17 departments, such as gynecology, pediatrics, internal medicine, and traditional Chinese medicine. Each department has a list of doctors. 

To maximize the representativeness of the sample, this study employed a random sampling method in February 2024. Random numbers were generated to collect and integrate text from the comment sections of doctors, randomly selecting three doctors from the lists of 17 departments. Comments that were overly simplistic or vague (e.g., “satisfied”, “thank you, doctor”, “bad”), highly repetitive due to template usage, or containing inappropriate language were excluded. Following this filtration process, a total of 2541 patient comments were obtained as the primary data for this study.

### 2.2. Methods 

#### 2.2.1. Grounded Theory

**Figure 1:**
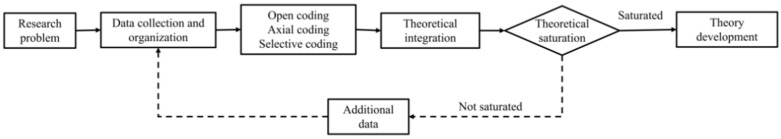
Research process based on grounded theory.

Grounded theory is one of the qualitative analysis methods, requiring researchers to fully utilize their theoretical acumen. Unlike traditional approaches, it does not begin with a theoretical hypothesis. Instead, data are collected from the real world based on research questions. The raw data are then abstracted and conceptualized, leading to the induction of a theoretical or conceptual framework. This approach is a classic bottom–up research method [32]. The process primarily involves three stages: open coding, axial coding, and selective coding (Figure 1).

Constructing a system of factors influencing patient satisfaction in the context of online healthcare requires a process that transitions from the figurative to the abstract. Such a system cannot be built solely on quantitative indicators or advanced personal experience. Grounded theory, however, facilitates the construction of a new theoretical framework based on empirical data within a specific context through a process of three-tier coding and induction. To ensure the reliability of grounded-theory coding and mitigate subjective bias, it is essential to employ multiple coders [33]. The original data were independently coded by a three-member coding team using NVivo 12, a grounded-theory analysis software. Following the independent coding process, the team convened regularly to reach a consensus. The Kappa method was used to assess inter-coder reliability, with a Kappa value of ≥0.75 indicating good consistency [34]. This approach aimed to minimize potential biases and ensure consistency in the interpretation of the data.

Open coding is the initial step in data analysis. During the open-coding process, the patients’ review text is organized. Through repeated readings, each sentence of the review serves as the unit of analysis for coding. The original sentences are dissected and summarized to obtain the initial concepts related to the theme. Subsequently, the concepts are sorted and refined, resulting in 23 categories. 

Second, axial coding is employed to identify the connections between the independent categories identified in the open-coding process by summarizing the categorization concepts and contents and establishing relationships among the categories through cluster analysis. This step helps in organizing and summarizing to form 12 main categories.

Third, selective coding further integrates and condenses the results of axial coding to reveal core categories, thereby focusing the analysis on the concepts and categories related to this core category. Through the analysis of the 23 categories and 12 main categories, this study summarizes patient satisfaction on the OMC platform into three core categories: audience perception of online health information, online health information provider performance, and online health information interaction channels.

Finally, the theoretical saturation test was employed to ascertain whether the grounded-theory research process could conclude sample collection. Five doctors who had not been previously selected were randomly chosen from the Chunyu Doctor app, and the text content of their patient comment area was utilized as test data. Here, 253 valid comments were extracted, and three-level coding and analysis were conducted. However, no new categories emerged, and no additional concepts were identified within the existing categories, indicating theoretical saturation had been reached. The constructed system of influencing factors for patient satisfaction on the OMC platform is shown in Table 1.

#### 2.2.2. DEMATEL

After identifying the influencing factors through grounded theory, it is equally important to discern the critical factors among them. Grounded theory provides a foundation for discovery, while the DEMATEL method serves as a tool for analysis and prioritization. DEMATEL is called the multi-criteria decision-making method. As a quantitative research method that has been widely used in recent years in the field of social sciences, it applies the experience and wisdom of experts to quantitatively analyze the relationship between various influencing factors in the quantitative indicator system and can identify and analyze the strength of the elemental relationships in a complex system and simplify the internal structure of the system [35,36,37,38]. In contrast to other decision-making methods such as AHP (analytic hierarchy process) and ANP (analytic network process), DEMATEL is particularly effective in identifying causal relationships within complex systems comprising multiple interacting factors. While AHP and ANP are valuable for prioritizing factors based on predefined criteria or network structures, DEMATEL excels in visualizing and quantifying causal relationships [39]. By constructing the direct influence matrix and then normalizing it to obtain the comprehensive influence matrix and then calculating the degree of influence, degree of being influenced, degree of cause, and degree of centrality of each influencing factor according to the comprehensive influence matrix, and drawing the causal diagram, we can identify and analyze the strengths of the elemental relationships in complex systems and find out the key factors that can solve the problem better, which can address complex and challenging decision-making issues in real-life situations. In this paper, we study the key influencing factors of patient satisfaction in OMC platforms, which requires careful consideration of the interactions between the influencing factors and the degree of importance of each influencing factor, making the DEMATEL method highly appropriate for this study.

An expert evaluation team was established to assess the influencing factors of patient satisfaction on the OMC platform. The expert survey primarily utilized a matrix-filling table, with the questionnaire survey serving as a supplementary. Prior to the survey, experts were provided with detailed explanations of the relevant concepts and instructions to ensure they could efficiently and comprehensively assign values to the relationships between the various influencing factors. A total of 52 respondents participated in the scoring process, consisting of eight academic experts from leading universities, such as Tsinghua University, Wuhan University, Nanjing University, Tianjin University, Sichuan University, Southwest Jiaotong University, Nanjing Agricultural University, and Jiangsu University. The selection of academic experts was based on their expertise in healthcare, decision-making methodologies, and information systems, ensuring a comprehensive understanding of the subject matter. Additionally, 44 experienced users of the OMC platform were included. To qualify as experienced users, the participants were required to have used the platform for at least two years and average a minimum of ten consultations per year. When scoring each influencing factor, the experts utilized a 5-point scale, employing a 0–4 range to assess the degree of influence [40] (with 4 indicating strong influence, 3 considerable influence, 2 moderate influence, 1 minimal influence, and 0 representing no impact). No invalid scoring questionnaires were submitted, resulting in the collection of 52 valid questionnaires.

## 3. Results

### 3.1. Establish and Calculate the Matrixes

By obtaining the scores of 52 experts on the degree of mutual influence between the factors affecting patient satisfaction on OMC platforms and integrating the experts’ ratings of the influence degree between various factors, a direct relationship matrix of order n was constructed. Each value in the matrix represents the degree of influence between the different factors, as shown in Formula (1). The data are homogenized to obtain the initial direct relation matrix.

(1) $$M=\left[\begin{array}{cccc} 0 & A_{12} & \cdots & A_{1j}\\ A_{21} & 0 & \cdots & A_{2j}\\ \vdots & \vdots & \ddots & \vdots \\ A_{i1} & A_{i2} & \cdots & 0 \end{array}\right]$$

Based on the establishment of the initial direct relationship matrix M, the column maximum method is used to normalize the direct relationship matrix. Specifically, the sum of the elements in each column is calculated to obtain the maximum value. Since there are cases where the sums of the elements in each column are equal, which may affect the calculation results, the sum of the elements in each corresponding column is compared with the sum of the elements in the corresponding row, and the smaller value is selected. Each element in matrix M is then divided by the selected smaller value. The normalized direct relationship matrix N is obtained by applying this process to the initial direct relationship matrix M, as shown in Formula (2).

(2) $$N=min\left[\frac{1}{max\sum_{i=1}^{n}A_{ij}},\frac{1}{max\sum_{j=1}^{n}A_{ij}}\right]M$$

**Figure 2:**
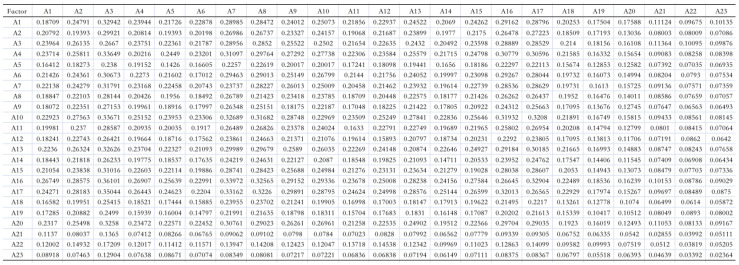
The total relation matrix T.

The multiplication of the normalized direct relation matrix N represents the increased indirect effects of each influencing factor. To illustrate the direct and indirect influencing relationships among the factors affecting patient satisfaction on the OMC platform, all indirect influences were calculated using MATLAB 2014a software according to Formula (3). The sum of these indirect influences yields the total relation matrix T  of factors influencing patient satisfaction on the OMC platform (Figure 2).

(3) $$T=\underset{n\to \infty }{\lim}\left(N+N^{2}+\ldots +N^{n}\right)={N(I-N)}^{-1}$$

I is the identity matrix; ${\left(I-N\right)}^{-1}$ is the inverse matrix of (I−N).

### 3.2. Calculate the Influence Degree, Affected Degree, Centrality, and Causality Degree

The influence degree ($D_{i})$ refers to the sum of the row values of the total relation matrix T, indicating the extensive influence of this factor on all other factors. The affected degree ($C_{i}$) is the sum of the values in each column, indicating the degree of comprehensive influence of this factor by all other factors. Centrality ($F_{i}$) is the sum of the influence degree ($D_{i}$) and affected degree ($C_{i}$), indicating the position and importance of factors in the index system. The causality degree ($R_{i}$) emphasizes the attribute of the factor and is obtained by subtracting the two. If the causality degree is greater than zero, it indicates that the factor significantly impacts other factors and is a causal factor. Otherwise, it is a resultant factor. The calculation formulas are shown in (4) to (7).

(4) $$D_{i}=\sum_{j=1}^{n}A_{ij},\;i=1,\;2,\;\cdots ,\;23$$

(5) $$C_{i}=\sum_{i=1}^{n}A_{ij},\;j=1,\;2,\;\cdots ,\;23$$

(6) $$F_{i}=D_{i}+C_{i},\;(i=1,\;2,\;\ldots ,\;23)$$

(7) $$R_{i}=D_{i}-C_{i},\;(i=1,\;2,\;\ldots ,\;23)$$

Based on the data in the total relation matrix and combined with Formulas (4)–(7), the influence degree, affected degree, centrality, and causality degree of each influencing factor was calculated (Table 2).

**Figure 3:**
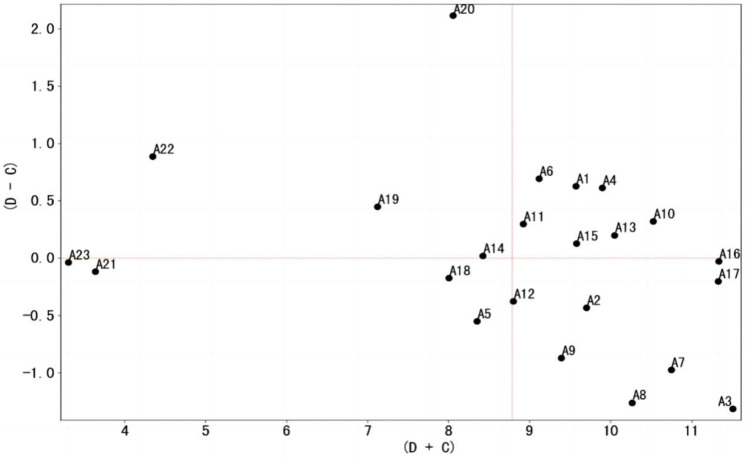
Cause–effect relationship diagram of influencing factors of patient satisfaction on the OMC platform.

To visually depict the influence of each factor, the centrality values of the factors were plotted on the *x*-axis, while the causality values were plotted on the *y*-axis. The specific positions of each influencing factor in the coordinate system were marked according to their coordinates (Figure 3). In this figure, the factors above the *X*-axis are causal factors, totaling 11, while the factors below the *X*-axis are resultant factors, totaling 12.

### 3.3. Results of the Identification of Key Influencing Factors

To facilitate further analysis, the values were arranged and ranked in descending order based on their influence degree, affected degree, causality degree, and centrality degree. The results are presented in Table 3. The key influencing factors in the system should be significant and valuable from a global and comprehensive perspective. Therefore, the author adopted the processing methods from related research [41], considering the four rankings—each factor’s influence, affected, centrality, and causality degrees—to determine the critical factors influencing patient satisfaction on the OMC platform. Specifically, the rankings of the four values associated with each factor are aggregated and subsequently averaged to derive the overall ranking score. Given that a lower score corresponds to a higher ranking, the factors are organized in ascending order based on their overall ranking scores to facilitate clearer visualization. The top 50% of factors—defined as those with comprehensive scores falling within the lower 50% of the total number of influencing factors (in this study, this threshold is 11.50)—are identified as the critical factors. The calculation results are shown in the last three columns of Table 3. From the table, the key influencing factors are: A16 (doctors provide professional treatment plans), A10 (doctors accurately understand patients’ concerns), A17 (doctors explain and advise on prescriptions), A4 (doctors personally respond), A13 (doctors provide comprehensive replies), A3 (cost-effectiveness), A1 (consultation fees), A7 (effectiveness of treatment outcomes), A6 (reasonableness of the doctors’ consultation process), A15 (avoidance of templated responses by doctors), and A8 (alignment of doctors’ responses with patient expectations). The comprehensive scores of these 11 factors do not exceed 11.50. The remaining 12 factors, with composite scores higher than 11.50, are not included as key influences. This conclusion is also evident in the individual rankings of these factors with respect to influence, affectedness, centrality, and causality. The 11 factors either do not exhibit a distinct individual ranking or display two or more rankings that are comparatively lower, which renders them unsuitable for designation as key influences.

## 4. Discussion

**Figure 4:**
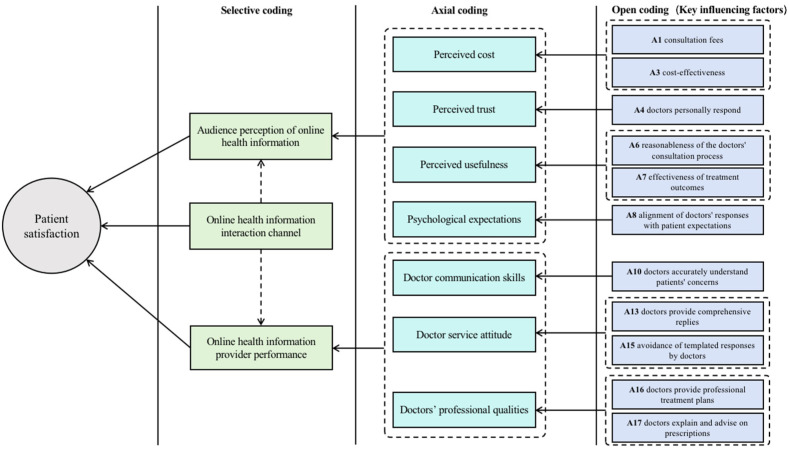
Visualization of key influencing factors of patient satisfaction on the OMC platform.

Motivating patients to actively engage with online medical consultation (OMC) platforms, retaining existing users, and attracting new ones are essential for the successful operation of these platforms and for maximizing the effectiveness of online medical consultations. To achieve this, healthcare providers and platform operators must identify the critical challenges among the various factors influencing patient satisfaction on these platforms. They must prioritize the most significant influencing factors and strategically promote them to enhance overall patient satisfaction. To better illustrate these key factors, their visualization is presented in Figure 4.

### 4.1. Doctors’ Professionalism 

The identification of 11 key influencing factors reveals that, throughout the online consultation process, the professionalism, communication skills, and service attitude of doctors are of utmost importance to patients. Professionalism remains the cornerstone of medical practice, requiring that doctors maintain the same standards in online consultations as in traditional, in-person consultations. Physicians must consistently uphold their responsibilities and professionalism. The process of pre-consultation questioning is crucial, as it reflects the physician’s genuine concern for the patient’s needs and issues. During this stage, the physician should analyze the underlying causes of the patient’s condition, employing refined diagnostic techniques to thoroughly understand the patient’s situation. Subsequently, the physician provides a detailed explanation of the condition, along with targeted diagnoses, recommendations, and treatment prescriptions, clarifying the potential benefits and implications of the suggested treatments. Emphasis must be placed on the efficiency of doctor–patient health information exchange, ensuring the delivery of relevant, patient-specific information while avoiding standardized responses. The factor “A13: doctors provide comprehensive replies” highlights that the more thorough and comprehensible the physician’s responses to the patient’s provided health information, the higher the patient’s satisfaction will be. This underscores the necessity for physicians, beyond technical aspects such as diagnostic accuracy and treatment efficacy, to emphasize non-technical elements, including humanistic care. This entails ensuring timely, friendly communication and mastering effective patient interaction, thereby making patients feel respected and valued, ultimately enhancing their satisfaction with online medical care.

### 4.2. Patients’ Engagement 

Research has demonstrated that inadequate doctor–patient communication is a major contributor to patient dissatisfaction. Patients frequently report issues with physicians’ communication abilities, often feeling that the information provided was either not comprehensible or did not address their queries adequately. However, prior studies have shown that, when doctors offer both informational and emotional support, it positively impacts patient satisfaction. Furthermore, the active engagement of patients has been found to enhance the likelihood of doctors providing such support [42,43]. In other words, patient proactivity can encourage physicians to enhance the efficiency of medical consultation services and assist in delivering more professional and accurate treatment plans. For instance, if patients actively engage in the consultation process, adequately prepare beforehand, compile relevant information through formal channels, and articulate their needs clearly and comprehensively, the efficiency of health information exchange with doctors can be improved to a certain extent.

In the traditional offline medical model, patients typically choose hospitals or physicians with strong medical reputations due to their limited medical knowledge [44]. Although the OMC platform provides patients with the autonomy to select their doctors, many still display a herd mentality, opting for renowned doctors from tertiary hospitals in major cities or those with high reputations at the time of consultation. This often results in elevated expectations of the doctor before the consultation. In practice, however, these patients may end up consulting doctors who are either unsuitable for their specific needs or fail to meet their expectations, thereby creating a gap between their anticipated and actual experiences.

Notably, the higher consultation fees charged by certain specialist physicians do not invariably ensure superior-quality responses, which can significantly diminish patient satisfaction. From the platform’s perspective, the implementation of recommender systems can enhance the visibility of less prominent physicians. These systems enable patients to filter their choices based on various criteria, including region, gender, and specialty, thereby facilitating the identification of physicians who more closely align with their specific needs [45].

### 4.3. The OMC Platforms’ Role

The fact that factors related to the information interaction channel have not emerged as key influences does not diminish the critical role of the OMC platform as a communication bridge between doctors and patients. While patients may outwardly express dissatisfaction with doctors’ performance, the underlying issue often lies in the imperfect mechanism of the platform [46]. The OMC platform needs to control the quality of the contracted doctors strictly. This involves strengthening the verification of doctors’ credentials during registration, including the addition of an online evaluation link, setting a threshold, and assessing professional knowledge and moral integrity.

Moreover, the platform should implement facial verification protocols for physicians prior to login, ensuring that they personally respond to patient inquiries, thereby reinforcing their professionalism and authority [47]. To further enhance physicians’ communication skills and service demeanor, the platform can offer targeted online training modules, focusing on areas such as text-based communication and response strategies. This approach enables physicians to effectively convey their professional knowledge to patients.

Given that online medical consultation (OMC) operates as a paid health information service, the study results indicate that both the pricing and perceived value of the service significantly impact patient satisfaction. Therefore, it is essential for the platform to regulate pricing structures, establish reasonable fee standards, and maintain a balanced comparison of prices for similar services available both online and offline, taking into account service categories and physician qualifications. Notably, the study highlights that comprehensive cost-effectiveness—emphasizing the value of services—holds greater importance to patients than the service fee itself, thereby surpassing the consultation fee in overall significance.

## 5. Conclusions

Doctor reviews on OMC platforms that are generated by patients reflect their needs and desires, providing comprehensive insights. This study initially employs the grounded-theory method to construct a system of factors influencing patient satisfaction with the OMC platform based on doctor reviews from the “Chunyu Doctor” app. This system encompasses 23 factors across three dimensions: audience perception of online health information and the performance of online health information providers. Subsequently, the DEMATEL method is utilized to identify 11 key influencing factors.

However, there remains considerable scope for further exploration of the factors influencing patient satisfaction with OMC platforms. Specifically, differences between patient categories warrant additional investigation. Patient groups with varying levels of health literacy, frequency of platform use, or specific health conditions may experience and prioritize satisfaction factors in distinct ways. Furthermore, given the diversity in functional design and regulatory settings across OMC platforms, this study focuses exclusively on the “Chunyu Doctor” app, the largest OMC platform in China. While the conclusions drawn are representative, future research should explore the applicability of these findings across a broader range of OMC platforms to enhance the generalizability of the results.

## Figures and Tables

**Table 1:** Influencing factors of patient satisfaction on the OMC platform.

Selective Coding	Axial Coding	Open Coding	Excerpts
Audience perception of online health information	Perceived cost	A1 consultation fees	Everything else is fine, but the consultation fee is high!
		A2 time cost	My child has a fever in the middle of the night, and there is nothing I can do. Come here to find a doctor for help. There are doctors online!
		A3 cost-effectiveness	Spending tens of yuan to ask for help is too low cost-effective. It is better to go to the hospital directly. Online consultation is a lie.
	Perceived trust	A4 doctors personally respond	I suspect that many doctors in Chunyu answer my questions on behalf of others. Even the typos in their answers are the same as those of others.
		A5 doctors’ patient reviews are reliable	I don’t know where you got so many good reviews. And 39 years of clinical experience?
	Perceived usefulness	A6 reasonableness of the doctors’ consultation process	Without careful examination of the early stage of the disease, many symptoms were diagnosed before being explained, and I felt that the diagnostic process was useless.
		A7 effectiveness of treatment outcomes	I took the medicine according to the doctor’s instructions and was fine.
	Psychological expectations	A8 alignment of doctors’ responses with patient expectations	We are in a small place, and the doctors are not very good. Nowadays, when children have problems, they go to the hospital for testing and then come to the Chunyu Doctor platform to see the results. Dr. Li is excellent.
Online health information provider performance	Doctor communication skills	A9 doctors’ explanation is understandable	Just like that, the doctor is too lazy and likes to speak. He can’t understand some of them and doesn’t know what he is suggesting.
		A10 doctors accurately understand patients’ concerns	He didn’t understand my point very well, so I asked Lonely.
	Doctor service attitude	A11 doctors responded proactively	Every question I asked had to be asked several times before I got a clear answer.
		A12 doctors’ response speed	Each message was spaced more than half an hour apart, from the first reply to me at around 3 p.m. to the end of the entire consultation at around 7 a.m. the following day.
		A13 doctors provide comprehensive replies	Just go back and have surgery. I was already undergoing surgery. I came to an online consultation just to understand the disease more comprehensively.
		A14 number of words in the doctors’ replies	The answers were all given in an understatement. Whenever I asked many questions, the doctor only replied with one sentence.
		A15 avoidance of templated responses by doctors	Dr. Lu answered questions very quickly, but it is evident that he used templates to answer common questions. After looking through multiple records, he found that the answers to the same disease were identical. I asked a total of four questions, and the answers to three of them were the same as his previous answers. Reply to others the same way.
	Doctors’ professional qualities	A16 doctors provide professional treatment plans	The content of the reply was found on Baidu. There was no professional reply, and it was no help to me. The doctor’s reply was not systematic, vague, and did not fully explain the diagnosis.
		A17 doctors explain and advise on prescriptions	The doctor’s reply was not systematic, vague, and did not fully explain the diagnosis results.
	Doctor’s empathy	A18 doctors show humanistic care	The doctor cannot understand the mood of the patient’s family and uses a rhetorical or accusatory tone.
Online health information interaction channel	Judgment mechanism for the end of consultation	A19 reasonableness of platform end consultation mechanism	This software is not particularly good. It says it can hold 50 conversations. I closed it before I finished using it.
	Platform doctor matching mechanism	A20 platform department and doctor matching	I initially consulted a neurologist, but somehow, he transferred me to a medical cosmetology department.
	Communication and feedback function design	A21 well-established platform refund mechanism	I searched for a long time but couldn’t find a refund! Simply speechless.
		A22 platform reply reminder feature works well	No reply prompt was received, and the consultation was closed...
	Platform service reliability	A23 platform user privacy protection	After asking questions on the platform, someone called me a few days later to say there was an unrestricted free clinic interaction. I wondered if the information had been leaked.

**Table 2:** The influence degree, affected degree, centrality, and causality degree for each factor.

Factor	Influence Degree	Affected Degree	Centrality Degree	Causality Degree
A1	5.10039	4.47422	9.57461	0.62617
A2	4.6343	5.07006	9.70436	−0.43576
A3	5.098	6.41191	11.50991	−1.31391
A4	5.25638	4.64344	9.89982	0.61294
A5	3.90132	4.45079	8.35211	−0.54947
A6	4.9035	4.21142	9.11492	0.69208
A7	4.88584	5.8612	10.74704	−0.97536
A8	4.5033	5.76409	10.26739	−1.26079
A9	4.25927	5.13089	9.39016	−0.87162
A10	5.42343	5.10156	10.52499	0.32187
A11	4.60693	4.31166	8.91859	0.29527
A12	4.21157	4.5875	8.79907	−0.37593
A13	5.12447	4.92431	10.04878	0.20016
A14	4.2196	4.20268	8.42228	0.01692
A15	4.85403	4.72479	9.57882	0.12924
A16	5.65436	5.68444	11.3388	−0.03008
A17	5.56338	5.76479	11.32817	−0.20141
A18	3.91238	4.08814	8.00052	−0.17576
A19	3.78629	3.33662	7.12291	0.44967
A20	5.08356	2.96848	8.05204	2.11508
A21	1.76228	1.87874	3.64102	−0.11646
A22	2.61559	1.72926	4.34485	0.88633
A23	1.6351	1.67428	3.30938	−0.03918

**Table 3:** Rank and criticality analysis of the influence degree, affected degree, cause degree, and centrality degree of influencing factors.

Influence Degree Analysis		Affected Degree Analysis		Causality Degree Analysis				Centrality Degree Analysis		Analysis of Key Influencing Factors		
Factor	Rank	Factor	Rank	Factor	Rank	Cause	Result	Factor	Rank	Factor	Score	Key?
A16	1	A3	1	A20	1	√		A3	1	A16	5.00	√
A17	2	A7	2	A22	2	√		A16	2	A10	5.50	√
A10	3	A17	3	A6	3	√		A17	3	A17	6.00	√
A4	4	A8	4	A1	4	√		A7	4	A4	7.00	√
A13	5	A16	5	A4	5	√		A10	5	A13	7.50	√
A1	6	A9	6	A19	6	√		A8	6	A3	8.00	√
A3	7	A10	7	A10	7	√		A13	7	A1	8.50	√
A20	8	A2	8	A11	8	√		A4	8	A7	9.25	√
A6	9	A13	9	A13	9	√		A2	9	A6	10.25	√
A7	10	A15	10	A15	10	√		A15	10	A15	10.25	√
A15	11	A4	11	A14	11	√		A1	11	A8	11.50	√
A2	12	A12	12	A16	12		√	A9	12	A20	11.75	×
A11	13	A1	13	A23	13		√	A6	13	A2	11.75	×
A8	14	A5	14	A21	14		√	A11	14	A11	12.50	×
A9	15	A11	15	A18	15		√	A12	15	A9	13.25	×
A14	16	A6	16	A17	16		√	A14	16	A14	15.00	×
A12	17	A14	17	A12	17		√	A5	17	A12	15.25	×
A18	18	A18	18	A2	18		√	A20	18	A19	16.25	×
A5	19	A19	19	A5	19		√	A18	19	A22	16.50	×
A19	20	A20	20	A9	20		√	A19	20	A5	17.25	×
A22	21	A21	21	A7	21		√	A22	21	A18	17.50	×
A21	22	A22	22	A8	22		√	A21	22	A21	19.75	×
A23	23	A23	23	A3	23		√	A23	23	A23	23.00	×

? indicates whether it is a key influencing factor, √ indicates yes, and × indicates no.

## Data Availability

The data presented in this study are available on request from the corresponding author.
